# The Associations between Blood and Urinary Concentrations of Metal Metabolites, Obesity, Hypertension, Type 2 Diabetes, and Dyslipidemia among US Adults: NHANES 1999–2016

**DOI:** 10.1155/2021/2358060

**Published:** 2021-10-25

**Authors:** Sarah Swayze, Michael Rotondi, Jennifer L. Kuk

**Affiliations:** School of Kinesiology and Health Science, York University, Toronto M3J 1P3, Canada

## Abstract

**Background:**

Heavy metals are well known to be associated with cancer outcomes, but its association with obesity and cardiometabolic risk outcomes requires further study.

**Methods:**

Adult data from the National Health and Examination Survey (NHANES Continuous 1999–2016, *n* = 12,636 to 32,012) with data for blood or urinary metals concentrations and body mass index were used. The study aim was twofold: (1) to determine the association between heavy metals and obesity and (2) to examine the influence of heavy metals on the relationship between obesity and hypertension, type 2 diabetes, and dyslipidemia. Logistic regression was used to examine the main effects and interaction effects of metals and obesity for the odds of prevalent hypertension, type 2 diabetes, and dyslipidemia. Models were adjusted for age, gender, ethnicity, smoking status, physical active status, and poverty-income ratio, with additional adjustment for creatinine in models with the urinary measures of heavy metals. High-low concentration categories were defined by grouping metal quintiles with the most similar associations with obesity.

**Results:**

Blood lead had a negative linear association with obesity (odds ratio (OR)  = 0.42, 95% confidence interval (CI) = 0.37–0.47). In those with obesity, high blood lead was associated with lower risk of prevalent dyslipidemia, while no association was found in those without obesity. The study observed a curvilinear relationship between urinary antimony and obesity with the moderate group having the highest odds of obesity (OR = 1.36, 1.16–1.59). However, the relationship between urinary antimony and prevalent hypertension and dyslipidemia risk was linear, positive, and independent of obesity. While not associated with prevalent obesity risk, high urinary uranium was associated with 30% (*P*=0.01) higher odds for prevalent type 2 diabetes.

**Conclusions:**

The impact of environmental factors on obesity and health may be complex, and this study reinforces the heterogeneous relationship between various metals, obesity, and obesity-related metabolic diseases even at levels observed in the general population.

## 1. Introduction

Heavy metals are a group of inorganic elements found on the periodic table with high densities, atomic weights, or atomic numbers. There is no standard definition for categorizing heavy metals as the definition varies depending on the author and context [1]. Generally, heavy metals are naturally occurring [2] and used in many different industries such as the mining, agricultural, medical, and technological sectors [3]. The widespread use of heavy metals in industries have made them persistent environmental contaminants as heavy metals cannot be degraded or destroyed [4]. Occupational exposure to heavy metals is regulated in most of the Western world at the federal level by agencies such as the National Institute for Occupational Safety and Health and Occupational Safety and Health Administration. Exposure to heavy metals can occur through oral, dermal, and respiratory routes [2] with the potential for exposure being higher in those working in industries that use heavy metals. This exposure can be measured at the source as is common in occupational settings by an industrial hygienist [5] or after exposure using a variety of biomarkers such urine and blood [6, 7]. While the usage of some of these metals has reduced over the years, these metals persist in the environment and some metals like lead bioaccumulate and cause more subtle chronic health effects, even at lower exposure levels [8, 9]. Long studied metals like lead and cadmium have been associated with a range of health effects such as hepatic toxicity, hypertension, and other cardiometabolic conditions [9–16]. However, it is unclear whether these metals influence obesity and obesity-related health risk.

The prevalence of obesity in the United States has increased over the past twenty years [17], and obesity is a well-known risk factor for other cardiometabolic diseases such as hypertension, type 2 diabetes, and dyslipidemia [18]. Together, these diseases are a burden on the economy and the healthcare system [19]. The current focus of obesity research has been on the influence of health behaviors such as diet and exercise, but there are other less studied factors that may influence obesity and the related cardiometabolic conditions, making it prudent to examine whether there are health effects of environmental pollutants such as heavy metals at levels more commonly observed in the general public. While there is a significant amount of research into the occupational limits and risks associated with higher exposure to metals, there is little research into how these metals may be associated with obesity rates and obesity-related cardiometabolic risk factors at exposure levels more commonly observed in the general public.

Heavy metal poisoning is generally associated with weight loss not weight gain [20, 21]. In humans, prenatal lead exposure is associated with decreased birth weight [22, 23]. In a study of adults from the National Health and Examination Survey (NHANES 1999–2002), blood or urine lead concentration is negatively associated with body mass index and waist circumference [24, 25]. However, in mouse models, maternal exposure to lead is associated with male offspring with increased body weight [26], and lead exposure in adult male mice is also associated with increased body weight [27]. In humans, barium exposure is observed to be associated with higher body mass index and higher waist circumference, but this association has yet to be investigated in longitudinal studies [25]. Uranium and cadmium are also potentially related with obesity and are also known endocrine disruptors [28, 29]. In mouse models, cadmium is associated with an increase in the levels of thyroid-stimulating hormones. Elevated levels of thyroid-stimulating hormones can lead to hypothyroidism and subsequent weight gain and increased waist circumference [28, 30, 31]. However, in US adults examined in the National Health and Nutrition Examination Survey, cadmium exposure is negatively associated with body mass index and waist circumference [25]. Thus, the relationship between heavy metals and obesity requires further elucidation.

The intersection of metal exposure and obesity, and their associated health outcomes, remains unclear. Commonly, studies regarding metal exposure and health risk adjust for body mass index or waist circumference, but these studies do not specifically examine those with obesity as a separate population, which may mask associations that are unique to those with obesity. As the prevalence of obesity increases, it is imperative to examine how environmental exposures may affect this population [32]. For example, polycyclic aromatic hydrocarbon exposure is an example of an environmental pollutant that has a pattern of association with hypertension that differs between those with and without obesity [33]. In individuals without obesity, there was a linear association between PAH and hypertension, while within individuals with obesity, there is an inverted U-association wherein the highest risk is associated with the middle quintile for 3-fluorene [33]. This example illustrates how environmental exposures can have differential health risks between obesity categories. The objective of this study is to gain insights into the relationship between the blood and urine measures of heavy metal exposure and obesity and to determine if heavy metals influence the relationship between obesity, hypertension, type 2 diabetes, and dyslipidemia.

## 2. Materials and Methods

### 2.1. Participants

NHANES Continuous is a nationally representative cross-sectional survey conducted biannually in the United States from 1999 onward [34]. Each NHANES Continuous survey release includes approximately 10,000 individuals who were assessed on a variety of health factors at a mobile examination unit and home interviews during a two-year period. Participants were asked a variety of questions regarding their health, dietary information, demographics, and socioeconomic status. The examination portion of the survey consists of medical, dental, physiological, and laboratory tests taken in mobile examination centers [34]. Participants at least 20 years of age from the continuous National Health and Nutrition Examination Survey (NHANES Continuous) 1999–2016 with available metal metabolites data were included in this study. There were eight metals studied with only urinary measures (antimony, barium, cesium, uranium, molybdenum, thallium, tungsten, and cobalt). In addition, two metals (cadmium and lead) were examined using blood and urinary measures. Beryllium and platinum were omitted due to inconsistencies in the method of measurement over the survey years. The total sample before exclusions was 92,062. Participants were excluded from analysis if they had missing data for any of the variables of interest (metals, height, weight, hypertension, type 2 diabetes, dyslipidemia, age, gender, ethnicity, poverty-income ratio, physical activity status, smoking status, and dietary caloric intake) (*n* excluded = 62,188 to 77,728), if they were pregnant or thought they might be pregnant (*n* excluded = 1897 to 2286), or if they had a BMI less than 18.5 kg/m^2^ (*n* excluded = 3345 to 15,322), depending on the metal examined. Individuals determined to be potential influencers and extreme outliers during univariate analysis were excluded from the analyses (*n* excluded = 7 to 19) [35, 36]. The final sample size ranged from 12,636 to 32,012, depending on the metal examined due to participant sampling differences between blood and urine measures, only those who met subsample requirements were eligible for urine sampling [6]. This is an analysis of publicly available data and does not require ethics approval from our institutional review board.

### 2.2. Survey Methods

Age, gender, ethnicity (white and other), poverty-income ratio, physical activity status (yes/no), smoking status (never, current, past), and dietary intake (calories) were obtained during the interview portion of the survey. The BMI was calculated from height and weight measured at the mobile examination centers. The cutoff for obesity was defined as 30 kg/m^2^ [37].

#### 2.2.1. Blood Metal Metabolites

Measures for blood cadmium and lead were collected during the mobile examination appointments by a trained phlebotomist [7, 38]. Samples were processed, frozen, and shipped to the National Center for Environmental Health for analysis. Blood cadmium and lead measures for survey years 1999–2002 were analyzed simultaneously using adapted methods from Miller et al. (1987), Parsons et al. (1993), and Stoeppler et al. (1980) [7]. Blood cadmium and lead (*n* = 32,012) measurement is based on the amount of light by ground-state atoms of cadmium and lead from either an electrodeless discharge lamp (EDL) or a hollow cathode lamp (HCL) source. Blood samples were diluted with a matrix modifier [39]. Blood concentrations for all other survey years were determined using inductively coupled plasma mass spectrometry. In accordance with NHANES lab procedures, diluted whole blood samples are converted into a spray using a nebulizer and then inserted into a spray chamber. The sample then passed through an area of plasma, and the mass spectrometer detected the ions at each mass. The resulting electrical signals from the ions are processed to show the concentration of the element [38].

#### 2.2.2. Urinary Metal Metabolites

Urine samples were collected from participants for analysis for antimony (*n* = 12,280), barium (*n* = 12,256), cadmium (*n* = 12,262), cesium (*n* = 12,362), lead (*n* = 12,362), uranium (*n* = 11,312), molybdenum (*n* = 12,435), thallium (*n* = 12,491), tungsten (*n* = 12,456), and cobalt (*n* = 12,525). Samples were processed, frozen, and shipped to the National Center for Environmental Health for analysis. Urine concentrations were measured using inductively coupled plasma mass spectrometry [6]. In accordance with NHANES lab procedures, liquid samples were converted into a spray using a nebulizer and then inserted into a spray chamber. The sample then passed through an area of plasma, and the mass spectrometer detected the ions at each mass. The resulting electrical signals from the ions are processed to indicate the concentration of the element [6].

#### 2.2.3. Hypertension, Type 2 Diabetes, and Dyslipidemia

Hypertension was defined as having an average systolic pressure of 130 mmHg or higher, an average diastolic pressure of 85 mmHg or higher [40], or use of a hypertension medication. Blood was drawn by a trained phlebotomist and shipped frozen to the University of Minnesota for analysis of glucose and lipid profiles [41]. Type 2 diabetes was defined as fasting plasma glucose levels greater than 7.0 mmol/L or A1C ≥ 6.5% or an oral glucose tolerance test ≥11.1 mmol/L or use of a diabetes medication [14]. Dyslipidemia was defined as having serum triglyceride levels ≥2.06 mmol/L, total cholesterol ≥6 mmol/L, HDL <1.04 mmol/L for men and <1.29 for mmol/L for women, or use of a cholesterol medication [42]. Medication data were compiled from the prescription drug questionnaire taken during the home interview [43].

### 2.3. Statistical Analysis

Participant characteristics are presented by metal concentration category with differences between high-low groups examined using chi-square tests for categorical variables and *t*-tests for continuous variables. Metal concentrations are reported as the geometric means with standard error (SE). Continuous variables are presented as means with SE, and categorical variables as prevalence and SE.

Logistic regression was performed to determine the relationship between obesity and quintile groups for metal concentration while adjusting for age, gender, ethnicity, smoking status, physical active status, poverty-income ratio, and creatinine for the urine measures. High-low concentration categories were defined by grouping metal quintiles with similar associations with obesity upon visual inspection. In instances where the metals appeared to have no association or a linear association with obesity, the bottom 80% was defined as low and the remaining 20% were considered high. In cases where the relationship between metal concentration and obesity was curvilinear, the metal concentration categories were split into three groups. The low category was used as the reference group for all analysis.

Adjusted logistic regression analyses were performed to examine the relationship between obesity and metal concentration category with the prevalent odds for hypertension, type 2 diabetes, and dyslipidemia. Models included obesity and metal concentration main effect and interaction terms adjusting for age, gender, ethnicity, smoking status, physical active status, poverty-income ratio, and creatinine for the urine measures.

Due to the complex nature of the NHANES design, all statistical analyses were performed using SAS 9.4 survey procedures with domain statements and weighted to be representative of the United States population. Sample weights for the blood metal analysis were calculated using the mobile examination center weights, and those for the urine metal analysis were calculated using the metals subsample weights. Statistical significance was set at *P* ≤ 0.05.

## 3. Results

Participant characteristics are shown by urinary and blood metal concentrations in Table 1. Individuals in the high-metal-concentration groups tended to be younger than the low-metal-concentration groups (*P* < 0.001), while both high blood and urinary cadmium and lead groups were significantly older (*P* < 0.001). The high-concentration groups were more likely to be male and have a higher prevalence of current smokers.

**Figure 1:**
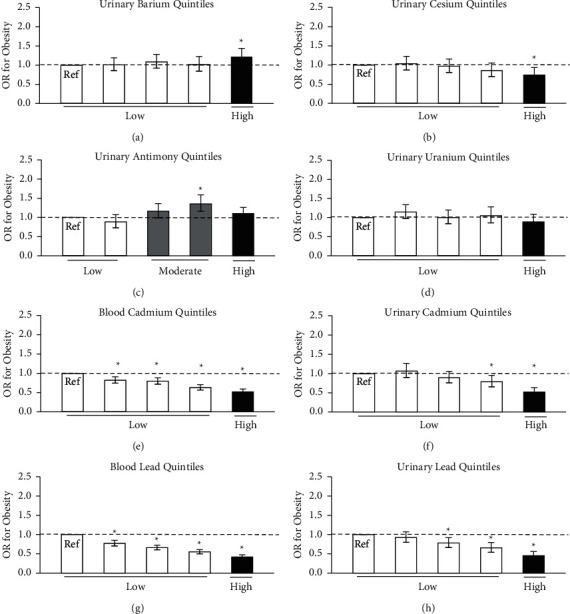
Odds ratio (OR) for obesity by heavy metal concentration quintile. White denotes low metal concentration, gray for moderate, and black for high. Models were adjusted for age, sex, poverty-income ratio, ethnicity, smoking status, urinary creatinine, calories consumed per day, and physical activity status. The error bars represent the 95% confidence interval for the odds ratio.  ^*∗*^Significantly different from the reference group, (*P* ≤ 0.05).

The relationship between metal concentration and obesity is outlined in Figure 1. A high concentration of urinary barium (OR 1.22, 95% CI: 1.04, 1.44) (Figure 1(a)) was positively associated with the odds of prevalent obesity. The relationship between obesity and urinary antimony was curvilinear with the high-concentration group having a similar association with obesity as the low-concentration group, with the moderate-concentration group having higher odds for prevalent obesity (OR = 1.36, 95% CI: 1.16, 1.59) (Figure 1(c)). There was a negative relationship between obesity and urinary cesium (OR = 0.74, 95% CI: 0.59, 0.94) (Figure 1(b)), urinary cadmium (OR = 0.52, 95% CI: 0.43, 0.63) (Figure 1(f)), urinary lead (OR = 0.45, 95% CI: 0.37, 0.56) (Figure 1(h)), blood cadmium (OR = 0.52, 95% CI: 0.46, 0.59) (Figure 1(e)), and blood lead (OR = 0.42, 95% CI: 0.37, 0.47) (Figure 1(g)). Molybdenum, thallium, tungsten, and cobalt were not associated with obesity or health (data not shown).

Table 2 presents the weighted prevalence of hypertension, type 2 diabetes, and dyslipidemia stratified by metal concentration category. High metal concentration was generally associated with lower prevalence rates of type 2 diabetes but higher prevalence rates of hypertension and dyslipidemia.

**Figure 2:**
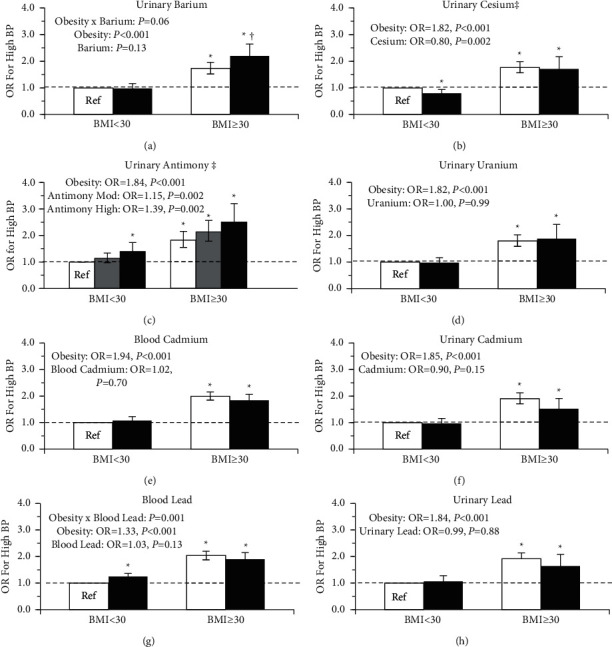
Odds ratio (OR) for hypertension by heavy metal concentration and obesity status. White denotes low metal concentration, gray for moderate, and black for high. Models were adjusted for age, sex, poverty-income ratio, ethnicity, smoking status, urinary creatinine, calories consumed per day, and physical activity status. The error bars represent the 95% confidence interval of the odds ratio.  ^*∗*^Significantly different from the reference group, (*P* ≤ 0.05). ^†^Metal group significantly different within BMI group, (*P* ≤ 0.05). ^‡^Significant metal main effect (*P* < 0.05). Obesity main effect significant in all models (*P* < 0.05).

Figure 2 outlines the associations between metal concentration, obesity, and prevalent hypertension with adjustment for age, sex, ethnicity, smoking status, poverty-income ratio, dietary intake, and physical activity. For hypertension, there was a trend for a significant interaction for urinary barium and obesity (Urinary Barium × Obesity, *P*=0.06), specifically obesity was associated with higher prevalent odds of hypertension, where the effect was larger in those with higher levels of urinary barium (Figure 2(a)). For hypertension, there was a significant interaction for blood lead and obesity (Blood Lead × Obesity, *P*=0.001), where high blood lead in those without obesity was associated with higher odds of hypertension but no difference in those with obesity (Figure 2(g)). Although the pattern of association between urinary lead and hypertension was similar to those with blood lead, the group differences for urinary lead failed to reach statistical significance (Urinary Lead × Obesity, *P*=0.09; Urinary Lead, *P*=0.88) (Figure 2(h)). A moderate concentration of urinary antimony was associated with a 15% higher odds ratio for hypertension, while a high concentration of urinary antimony was associated with 39% higher odds of hypertension, independent of obesity (Figure 2(c)). Conversely, a high concentration of urinary cesium was associated with 20% lower odds of prevalent hypertension independent of obesity (Figure 2(b)). For all other metals, there were no significant associations with hypertension (*P* > 0.05).

**Figure 3:**
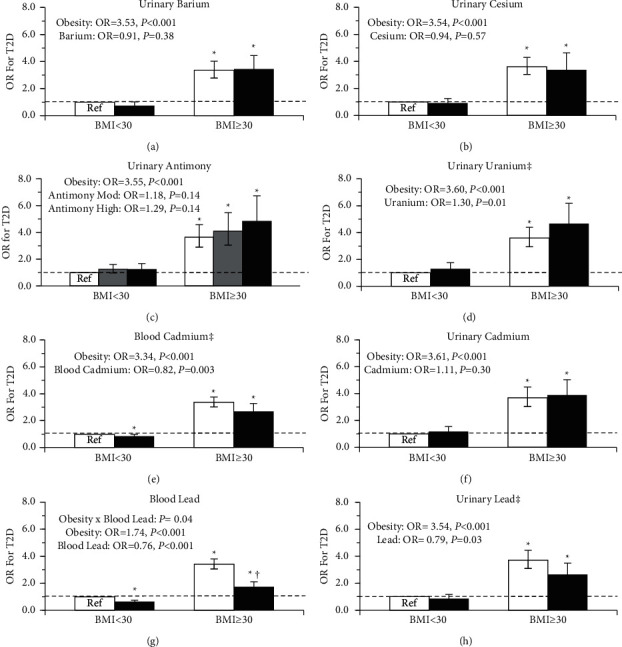
Odds ratio (OR) for type 2 diabetes by heavy metal concentration and obesity status. White denotes low metal concentration, gray for moderate, and black for high. Models were adjusted for age, sex, poverty-income ratio, ethnicity, smoking status, urinary creatinine, calories consumed per day, and physical activity status. The error bars represent the 95% confidence interval of the odds ratio.  ^*∗*^Significantly different from the reference group, (*P* ≤ 0.05). ^†^Metal group significantly different within BMI group, (*P* ≤ 0.05). ^‡^Significant metal main effect (*P* < 0.05). Obesity main effect significant in all models (*P* < 0.05).

Figure 3 outlines the associations between metal concentration, obesity, and prevalent type 2 diabetes with adjustment for covariates. High blood lead was associated with lower odds for type 2 diabetes, wherein the difference between blood lead groups was smaller in those without obesity (Blood Lead × Obesity, *P*=0.04) (Figure 3(g)). Similarly, urinary lead was associated with 21% lower odds of type 2 diabetes, but with no differences by obesity status (Figure 3(h)). High urinary uranium was associated with 30% higher odds of type 2 diabetes, independent of obesity (Figure 3(d)). Conversely, high blood measures of cadmium were associated with 18% lower odds of type 2 diabetes, independent of obesity (Figure 3(e)). Urinary cadmium and all other metals were not significantly associated with type 2 diabetes (*P* > 0.05).

**Figure 4:**
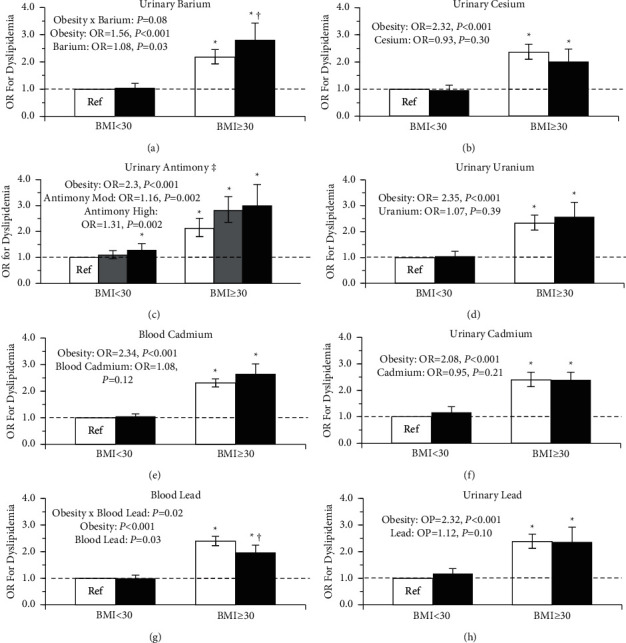
Odds ratio (OR) for dyslipidemia by heavy metal concentration and obesity status. White denotes low metal concentration, gray for moderate, and black for high. Models were adjusted for age, sex, poverty-income ratio, ethnicity, smoking status, urinary creatinine, calories consumed per day, and physical activity status. The error bars represent the 95% confidence interval of the odds ratio.  ^*∗*^Significantly different from the reference group, (*P* ≤ 0.05). ^†^Metal group significantly different within BMI group, (*P* ≤ 0.05). ^‡^Significant metal main effect (*P* < 0.05). Obesity main effect significant in all models (*P* < 0.05).

Figure 4 illustrates the associations between metal concentration, obesity, and prevalent dyslipidemia with adjustment for covariates. For dyslipidemia, there was a trend for a significant interaction for urinary barium and obesity (Urinary Barium × Obesity, *P*=0.08), wherein high barium was only associated with higher prevalent odds of dyslipidemia in those with obesity (Figure 4(a)). For dyslipidemia, there was a significant interaction for blood lead and obesity (Blood Lead × Obesity, *P*=0.02), wherein high blood lead was associated with lower odds of prevalent dyslipidemia in only those with obesity (Figure 4(g)). A moderate and high concentration of urinary antimony was associated with a 16% and 31% higher risk of dyslipidemia than low antimony, respectively, independent of obesity (Figure 4(c)). For all other metals, there was no evidence of significant associations with dyslipidemia (*P* > 0.05).

## 4. Discussion

This study examined the relationship between heavy metal concentration and obesity and whether metal concentration is associated with differences in how obesity relates with hypertension, type 2 diabetes, and dyslipidemia in the general population. This study observed positive, negative, and null relationships between metal concentration and obesity. Furthermore, metal concentration is associated with both better and worse health profiles for a given level of obesity. Thus, the impact of single factors on obesity and health may be complex and reinforces the hypothesis of a heterogeneous relationship between heavy metals, obesity, and metabolic disease.

The negative association between body weight and certain metals such as lead and cadmium has been established empirically [25, 44, 45]. Accordingly, this study also reports that metals such as lead, cadmium, and cesium were strongly negatively associated with obesity. However, this study and a previous report [25] suggest there may also be positive associations between barium and body mass index. While there was no significant relationship between obesity and uranium exposure in this study, a longitudinal study of Kuwaiti children suggests the relationship between obesity and higher levels of 2PY, a potential biomarker of uranium uptake [46]. To date the research on antimony exposure and obesity is limited [47]; however, this study observed a curvilinear relationship between antimony and obesity with the moderate-concentration group having the highest risk of obesity. This is in contrast to a previous report by Padilla et al. [25] showing no evidence of an association between antimony, BMI, and waist circumference. Thus, the direction and pattern of association between metals and obesity may differ depending on the metal in question and potentially the measure of obesity. Furthermore, it is important to remember that there may be other negative health effects associated with even the modest exposure to these metals that may also influence the association between obesity and cardiometabolic health effects.

While relatively little is reported regarding the association between some heavy metals and cardiometabolic risk factors, obesity is a well-known independent risk factor for several cardiometabolic risk factors. This study suggests that heavy metals may also be associated with cardiometabolic risk factors through differences in obesity but may also be an independent risk factor. Depending on the metal, there may be a positive or negative association with hypertension. For example, a higher incidence of hypertension was observed after occupational exposure to barium [48]. Similarly, this study suggests that high urinary barium was positively associated with prevalent hypertension, but only in those with obesity. This is consistent with previous literature in rats suggesting barium exposure adversely affects systolic blood pressure [49]. However, an older study published in 1981 reported no differences in hypertension rates in communities with high and low levels of barium in their drinking water [50]. This study did not report body mass index or obesity status and given that this study observed a greater effect of barium on hypertension in those with obesity; these differences may reflect the likely lower prevalence of obesity in this older study. Conversely, this study observed that across all obesity categories, cesium was associated with lower risk of prevalent hypertension. This is in contrast to Shiue [51] who reported a positive association between cesium and prevalent hypertension risk when using a substantively smaller data set of only the NHANES data from 2011 to 2012, while a small study from Brazil concluded those who were exposed to cesium had a prevalence of hypertension that was similar to that of the general population [52]. Lead exposure has long been known associated with weight loss and hypertension [16, 53]. However, this study demonstrates that the association between blood lead and hypertension may be dependent on obesity status. Consistent with past literature [54], this study demonstrates a positive association between high blood lead levels and hypertension in individuals without obesity; however, this study also demonstrated no association between high blood lead levels and hypertension in those with obesity. Reasons for these findings are unclear and warrant further investigation.

In terms of type 2 diabetes, only three metals demonstrate significant associations, but in differing directions. Consistent with a previous study using a smaller subset of NHANES data (1999–2010), this study showed that uranium is associated with higher prevalent diabetes risk [29]. In mouse models, even at low levels consistent with normal environmental exposure, uranium can act as an endocrine disruptor, which is a potential cause for type 2 diabetes, though the mechanism of action is as yet unknown [55, 56]. While the nephrotoxicity of uranium is well known [57], this study supports the notion of a potential link between uranium exposure and type 2 diabetes. Uranium exposure could cause a potential positive feedback loop between the exposure, type 2 diabetes, and kidney disease particularly in areas of high environmental contamination and high type 2 diabetes prevalence such as the Navajo Nation in the United States [58] and countries like Kuwait [46, 59] and thus increasing the healthcare burden of these communities. Conversely, this study demonstrates that high lead and cadmium were negatively associated with prevalent diabetes. The published research is conflicted [60–62]; however, it is interesting to note that in this study, both lead and cadmium were also negatively associated with obesity. Nevertheless, cadmium exposure even at these low levels may still cause kidney damage, which may complicate other health conditions such as type 2 diabetes [63–65]. Thus, obesity and type 2 diabetes risks may be associated with certain metal exposures, and metal exposures may also alter how obesity relates with type 2 diabetes.

There have been very few studies reporting the relationship between dyslipidemia risk and metal exposure. While our results show that while the relationship between antimony and obesity is curvilinear, there is a strong linear relationship with dyslipidemia. The health effects of barium are largely based on animal studies and the reported health effects include cardiovascular and metabolic disorders; however, little is known about its effect on cholesterol metabolism in the human body [66]. Interestingly, barium was associated with higher risk of prevalent dyslipidemia only in those with obesity, suggesting that metals such as barium may further exacerbate cardiometabolic risk in those with obesity. Conversely, this study suggests high blood lead levels are associated with lower risk of prevalent dyslipidemia in those with obesity. Lead exposure can play a part in pathway for cholesterol synthesis due to the hepatic toxic effects of lead [10]. Interestingly, higher levels of blood lead were also negatively associated with obesity. However, lead exposure in rats was observed to increase total serum cholesterol [67], and exposure at occupational levels were reported to be associated with higher total cholesterol and HDL cholesterol in those with and without obesity [68], suggesting the role of heavy metals and dyslipidemia may differ by dose and between lean populations and those with obesity.

There are several strengths and limitations worth mentioning. First, this study used a large data set weighted to be representative of the United States population. However, due to the cross-sectional nature of the survey design, causality cannot be established. The use of a single time point measure of urine and blood for the metals may not fully estimate participant exposure to these metals. In addition, it is difficult to determine what should be considered high vs. low exposure as there are no universal cutoffs for blood or urine measures for these outcomes. Furthermore, exposure to these metals is likely not in isolation and the combined effects of different metals may antagonize or exacerbate the effects of other metals, which given the heterogeneous associations observed may have biased our results toward the null. Also due to the anonymized nature of the data, it is not possible to determine the potential sources of exposure. Future longitudinal studies are needed to validate these findings.

## 5. Conclusions

In summary, this study used NHANES Continuous (1991–2016), a nationally representative survey to examine the associations between blood and urinary concentrations of heavy metals in the general population of the United States to determine whether heavy metals influence the cardiometabolic health effects associated with obesity. This study suggests that heavy metals and obesity have a potentially complex relationship with health profiles within the general population. This study observed negative, positive, and curvilinear associations between heavy metals and health risk factors within the general population of the United States. Furthermore, this association may differ by obesity status. This study highlights the need for further investigation into the cardiometabolic effects of environmental exposure of heavy metals at levels seen in the general public in addition to traditional cancer and neurodegenerative outcomes seen at higher exposure levels.

## Figures and Tables

**Table 1:** Participant characteristics by metal.

Weighted	Urinary barium		Urinary cesium		Urinary antimony			Urinary uranium	
	Low concentration	High concentration	Low concentration	High concentration	Low concentration	Moderate concentration	High concentration	Low concentration	High concentration
N	9818	2438	9860	2502	5100	4740	2440	9099	2213
Metal concentration, ng/ml^a^	0.88 (0.01)	4.76 (0.06)	3.3 (0.03)	10.3 (0.07)	0.03 (0.01)	0.08 (0.01)	0.23 (0.01)	0.004 (0.01)	0.028 (0.01)
Age, years	47.3 (0.3)	45.0 (0.4)^*∗*^	46.9 (0.3)	46.2 (0.4)	49.1 (0.4)	46.2 (0.3)	42.8 (0.4)^*∗*^	47.1 (0.3)	46.2 (0.5)
BMI, kg/m^2^	28.6 (0.1)	29.5 (0.2)^*∗*^	28.7 (0.1)	29.1 (0.2)^*∗*^	28.1 (0.1)	29.5 (0.1)	29.0 (0.2)^*∗*^	28.7 (0.1)	29.3 (0.2)^*∗*^
Obesity^b^, %	32.8 (0.7)	39.4 (1.3)^*∗*^	33.6 (0.7)	36.0 (1.3)^*∗*^	30.2 (0.9)	38.6 (1.0)^*∗*^	34.8 (1.3)^*∗*^	34.3 (0.8)	36.7 (1.5)
Sex, % male	47.7 (0.6)	55.8 (1.2)^*∗*^	48.2 (0.6)	54.5 (1.1)^*∗*^	43.6 (0.8)	51.5 (0.9)^*∗*^	58.8 (0.9)^*∗*^	48.5 (0.6)	54.2 (1.4)^*∗*^
White ethnicity, %	68.8 (1.2)	76.8 (1.2)^*∗*^	70.4 (1.2)	71.7 (1.4)^*∗*^	74.0 (1.3)	69.1 (1.4)^*∗*^	66.4 (1.7)^*∗*^	71.5 (1.2)	65.9 (2.3)^*∗*^
Current smoker, %	21.0 (0.6)	24.1 (1.2)^*∗*^	21.3 (0.6)	23.3 (1.0)^*∗*^	17.4 (0.7)	23.4 (1.0)^*∗*^	28.0 (1.2)^*∗*^	20.1 (0.5)	27.7 (1.3)^*∗*^
Intake, kcal/day	2153 (12)	2322 (24)^*∗*^	2182 (12)	2205 (25)	2128 (17)	2192 (18)^*∗*^	2341 (27)^*∗*^	2183 (12)	2226 (27)^*∗*^
Physically active, %	81.3 (0.6)	84.1 (0.9)^*∗*^	81.4 (0.6)	84.3 (0.9)^*∗*^	82.3 (0.8)	81.7 (0.8)	82.0 (1.0)	82.8 (0.6)	77.3 (1.2)^*∗*^

Weighted	Blood cadmium		Urinary cadmium		Blood lead			Urinary lead	
	Low concentration	High concentration	Low concentration	High concentration	Low concentration	High concentration		Low concentration	High concentration
N	25577	6435	9944	2318	25664	6348		9851	2511
Metal concentration, ng/ml^a^	2.4 (0.02)	11.2 (0.09)	0.16 (0.01)	1.01 (0.01)	0.05 (0.01)	0.18 (0.01)		0.4 (0.01)	1.8 (0.02)
Age, years	46.8 (0.2)	47.9 (0.3)^*∗*^	45.4 (0.3)	54.1 (0.4)^*∗*^	45.5 (0.2)	55.0 (0.3)^*∗*^		46.5 (0.3)	50.0 (0.4)^*∗*^
BMI, kg/m^2^	29.0 (0.7)	27.7 (0.1)^*∗*^	28.9 (0.1)	28.5 (0.2)	29.0 (0.1)	27.6 (0.1)^*∗*^		29.0 (0.1)	28.2 (0.2)^*∗*^
Obesity^b^, %	36.1 (0.5)	28.9 (0.7)^*∗*^	34.6 (0.7)	33.3 (1.3)	36.2 (0.5)	27.4 (0.8)^*∗*^		34.9 (0.7)	31.4 (1.3)^*∗*^
Sex, % male	50.0 (0.3)	48.4 (0.7)^*∗*^	50.2 (0.6)	46.1 (1.4)^*∗*^	46.0 (0.3)	69.4 (0.8)^*∗*^		46.9 (0.6)	62.9 (1.3)^*∗*^
White ethnicity, %	71.4 (1.1)	72.4 (1.4)^*∗*^	71.5 (1.2)	66.4 (1.6)^*∗*^	72.3 (1.1)	68.3 (1.5)^*∗*^		72.1 (1.2)	64.0 (1.6) ^*∗*^
Current smoker, %	9.5 (0.3)	75.0 (0.8)^*∗*^	18.3 (0.5)	39.8 (1.5)^*∗*^	19.8 (0.4)	33.6 (0.8)^*∗*^		19.8 (0.5)	31.0 (1.4)^*∗*^
Intake, kcal/day	2201 (9)	2202 (18)^*∗*^	2221 (12)	2035 (26)^*∗*^	2192 (10)	2247 (20)^*∗*^		2184 (12)	2231 (27)^*∗*^
Physically active, %	83.0 (0.4)	77.4 (0.7)^*∗*^	83.2 (0.6)	75.6 (1.1)^*∗*^	82.7 (0.4)	78.2 (0.7)^*∗*^		82.2 (0.6)	81.0 (1.0)

BMI, body mass index; kcal, kilocalories. For continuous variables, weighted mean (SE) are reported, and for categorical variables, weighted prevalence (SE) are reported. ^a^Metal concentration presented as geometric mean (SE). ^b^Obesity defined as BMI ≥30 kg/m^2^.  ^*∗*^*P* ≤ 0.05, different from low-concentration group.

**Table 2:** Weighted prevalence rates for hypertension, type 2 diabetes, and dyslipidemia by metal metabolite.

	Urinary barium			Urinary cesium	
	Low concentration	High concentration		Low concentration	High concentration
Hypertension, %	36.4 (0.6)	36.1 (1.2)		37.0 (0.6)	33.8 (1.1)^*∗*^
Type 2 diabetes, %	10.1 (0.4)	8.2 (0.6)^*∗*^		10.1 (0.4)	8.3 (0.7)^*∗*^
Dyslipidemia, %	52.6 (0.7)	57.2 (1.3)^*∗*^		53.3 (0.7)	54.7 (1.3)

	Urinary antimony			Urinary uranium	
	Low concentration	Moderate concentration	High concentration	Low concentration	High concentration
Hypertension, %	36.5 (0.9)	36.6 (1.0)	31.2 (1.3)	35.5 (0.7)	35.4 (1.4)
Type 2 diabetes, %	9.5 (0.5)	10.3 (0.5)	8.8 (0.7)	9.5 (0.4)	11.6 (0.9)^*∗*^
Dyslipidemia, %	50.1 (0.9)	55.6 (1.0)^*∗*^	57.4 (1.2)^*∗*^	51.9 (0.7)	55.9 (1.5) ^*∗*^

	Blood cadmium			Urinary cadmium	
	Low concentration	High concentration		Low concentration	High concentration
Hypertension, %	36.7 (0.5)	36.8 (0.8)		34.8 (0.6)	44.0 (1.3)^*∗*^
Type 2 diabetes, %	10.1 (0.3)	9.4 (0.4)		9.1 (0.3)	12.9 (0.8)^*∗*^
Dyslipidemia, %	52.2 (0.5)	58.2 (0.9)		52.2 (0.7)	61.1 (1.3)^*∗*^

	Blood lead			Urinary lead	
	Low concentration	High concentration		Low concentration	High concentration
Hypertension, %	34.3 (0.5)	49.4 (1.0)^*∗*^		35.4 (0.6)	41.2 (1.4)^*∗*^
Type 2 diabetes, %	9.9 (0.3)	10.2 (0.5)		9.7 (0.4)	9.7 (0.7)
Dyslipidemia, %	53.0 (0.5)	55.3 (0.9)^*∗*^		52.6 (0.7)	58.9 (1.3)^*∗*^

Weighted prevalence (SE) reported.  ^*∗*^*P* ≤ 0.05, different from low-concentration group.

## Data Availability

The NHANES data sets are publicly available (https://www.cdc.gov/nchs/nhanes/index.htm).
